# Functional Identification of Neuroprotective Molecules

**DOI:** 10.1371/journal.pone.0015008

**Published:** 2010-11-24

**Authors:** Cheng Dai, Dong Liang, Huiwu Li, Masayuki Sasaki, Ted M. Dawson, Valina L. Dawson

**Affiliations:** 1 Neuroregeneration and Stem Cell Programs, Institute for Cell Engineering, Johns Hopkins University School of Medicine, Baltimore, Maryland, United States of America; 2 Department of Physiology, Johns Hopkins University School of Medicine, Baltimore, Maryland, United States of America; 3 Department of Neurology, Johns Hopkins University School of Medicine, Baltimore, Maryland, United States of America; 4 Solomon H. Snyder Department of Neuroscience, Johns Hopkins University School of Medicine, Baltimore, Maryland, United States of America; Hungarian Academy of Sciences, Hungary

## Abstract

The central nervous system has the capacity to activate profound neuroprotection following sub-lethal stress in a process termed preconditioning. To gain insight into this potent survival response we developed a functional cloning strategy that identified 31 putative neuroprotective genes of which 28 were confirmed to provide protection against oxygen-glucose deprivation (OGD) or excitotoxic exposure to N-methyl-D-aspartate (NMDA) in primary rat cortical neurons. These results reveal that the brain possesses a wide and diverse repertoire of neuroprotective genes. Further characterization of these and other protective signals could provide new treatment opportunities for neurological injury from ischemia or neurodegenerative disease.

## Introduction

In response to sub-lethal insults, the central nervous system has the capacity to protect itself from subsequent serious injury by transiently activating the expression of survival networks. This form of neuronal plasticity, termed preconditioning is dependent on new gene transcription and protein translation [1]. Diverse stimuli ranging from spreading depression, transient ischemic episodes, hypoxia, anoxia, exposure to glutamate excitotoxins and cytokines can induce preconditioning. Gene activation and repression set in motion by these preconditioning stimuli is temporally specific and induces diverse families of pro-survival genes that enhance the brain's resistance to injury [1].

The nature of the preconditioning stimulus determines the transduction pathway that ultimately leads to post-translational modifications and/or activation of transcriptional responses that drive the genomic survival response. A number of transcription factors are thought to participate in the process and include cAMP response element-binding protein (CREB), myocyte enhancer factor 2 (MEF2), nuclear factor κB (NFκB), hypoxia-inducible factor (HIF) and others. While the neuroprotective molecules and networks that these events trigger are largely unknown, a consistent observation is that the brain's response to these stressors can subsequently provide profound protection against subsequent lethal injury [1].

Since this neuroprotective network requires new gene transcription and protein translation, it is highly suited for gene screening strategies. To gain insight into this neuroprotection we generated a cDNA library from preconditioned cortical neurons and conducted a functional cloning screen for protective molecules. Here we describe the results of this screen and the identification of 31 neuroprotective genes of diverse function.

## Results

### Establishment of Preconditioning

**Figure 1:**
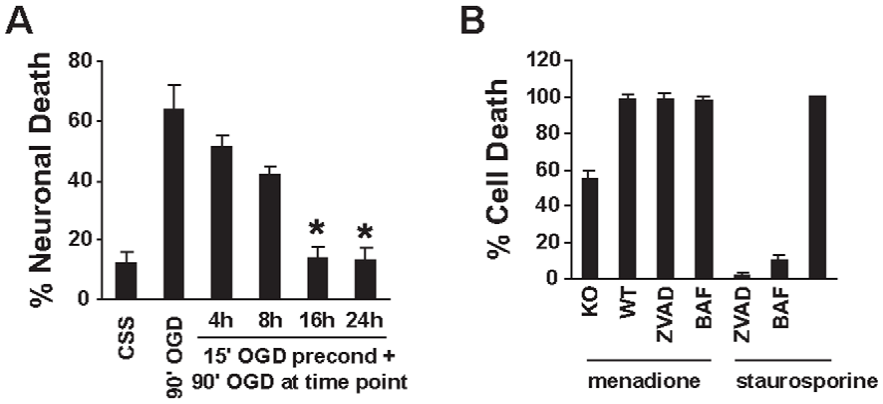
Preconditioning induces neuroprotection and menandione kills cells via Parthanatos. (A) 15 min of OGD preconditioning induces neuroprotection 16 h and 24 h later against 90 min of lethal OGD. Primary cortical cultures were exposed to 90 min lethal OGD without preconditioning (90′ OGD) and compared to cultures exposed to 15 min of OGD and rechallenged with 90 min OGD 4 h, 6 h, 16 h and 24 h after the 15 min of OGD preconditioning. Cell viability was determined 24 h after 90 min lethal OGD by Hoescht 33342 (1 µg/ml) and prodidium iodide (7 µM) staining and unbiased computer assisted cell counting [29]. (B). Menadione induces Parthanatos (PARP-1-dependent caspase-independent) fibroblast death. Menadione (175 µM, 10 min) kills almost all of the wild type fibroblasts (WT) but only 55% or PARP-1 knockout fibroblasts (KO). Menadione induced fibroblast death cannot be blocked by the caspase inhibitor, ZVAD (100 µM) or BAF (50 µM), which can blocks staurosporine (500 nM, 24 h) induced cell death. Experiments were performed four times. Data are the mean ± SEM. *p<0.01 (Student's t-test) comparing OGD (90 min) against OGD (15 min/90 min). An average number of 500,000 cells were counted.

Preconditioning that elicits survival sustainable for several days requires new gene transcription and protein translation. To identify the time point at which these genes are expressed and thus the optimal time point to harvest RNA to generate the cDNA library a time course for development of preconditioning was conducted. Cortical cultures were exposed to a preconditioning condition of 15 min OGD and then returned to growth media. At the indicated time points the cultures were challenged with a lethal exposure of 90 min OGD and 24 hrs later cell viability was evaluated. These experiments reveal that the protective effects of OGD preconditioning were clear and reproducible as early as 16 hr after OGD preconditioning (Fig. 1A).

### Conditions for functional screening in mouse fibroblasts

In the nervous system NMDA excitotoxicity is mediated largely by parthanatos, poly (ADP-ribose) polymerase 1 (PARP-1) dependent cell death [2], [3]. Since the functional screening strategy employed here requires dividing cells, it cannot be conducted in postmitotic neuronal cultures. Therefore cell death conditions in mouse fibroblasts that are consistent with PARthanatos, in which cells die in a PARP-1 dependent, caspase independent cell death pathway were developed. To reduce the number of false-positive surviving cells, conditions that killed 100% of the fibroblasts needed to be identified. 2-methyl-1,4-napthoquinone (menadione) is commonly used to induce oxidative stress and can kill cells through parthanatos. Exposure for 10 min to 175 µM menadione was found to kill 100% of wild-type fibroblasts, but only 55% PARP-1 knockout fibroblasts (Fig. 1B). This cell death cannot be blocked by the caspase inhibitors, ZVAD (100 µM) or BAF (50 µM). Mouse fibroblasts, both wild-type and PARP-1 knockout, exposed to a relatively non-selective protein kinase inhibitor, staurosporine (500 nM, 24 h) die in a caspase-dependent manner as ZVAD (100 µM) or BAF (50 µM) protect against staurosporine induced cell death (Fig. 1B). Therefore, exposure to menadione (175 µM, 10 min) kills cells via parthanatos, since wild-type fibroblasts die in a PARP-1-dependent but caspase-independent fashion.

### Identification of survival genes

**Figure 2:**
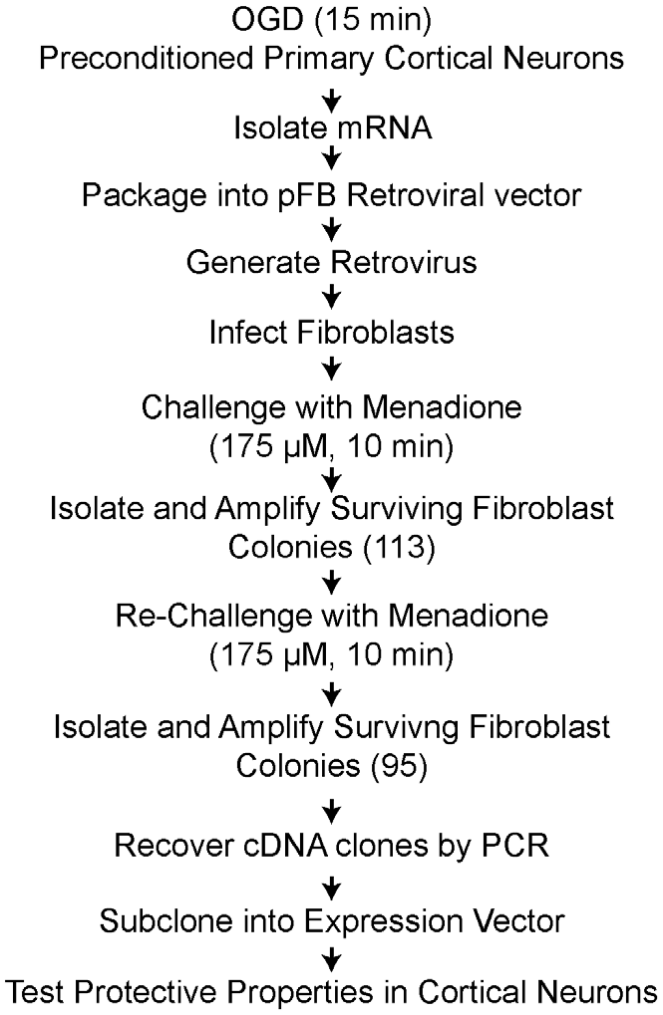
Functional cloning strategy. Diagram of the functional cloning strategy utilized to identify neuroprotective genes (see text for details).

To identify endogeneous brain protective genes, we generated a retroviral cDNA expression library from primary cortical neurons preconditioned with OGD and conducted a functional cloning screen for protective molecules (Fig. 2). The mRNA for the library was obtained from cortical neurons 16 h after a preconditioning stimulus of 15 min of OGD, which leads to sustained and profound protection against a subsequent lethal 90 min OGD treatment (see Fig. 1A). The cDNA library from OGD preconditioned cortical cultures with an average insert size of 2.5 Kb was cloned into the pFB retrovirus vector and virus was generated. Primary mouse fibroblasts were infected with the retrovirus and 48 h after infection the fibroblasts were challenged with menadione (175 µM, 10 min) to induce parthanatos. Cells that survived were grown another 9–13 days, and then re-challenged with menadione to confirm cytoprotection and eliminate false positives. 113 cell clones survived the initial menadione treatment. Of these, 95 survived the menadione re-challenge. The protective genes were recovered from the surviving cell clones by PCR with oligonucleotide primers that recognize regions flanking the multiple cloning site of the pFB vector (Fig. 2). A total of 31 independent genes were recovered and sequenced from the 95 cell clones (Table 1) Thirteen of the genes were designated neuroprotective genes (NGP1 to NPG13) and the sequences were deposited in the Genbank database. Accession numbers are indicated in Table 1.

**Table 1:** Cytoprotective Genes Identified by Functional Cloning.

Name	Accession number	# hits	Function
γ-Actin (cytoplasmic γ-isoform of actin)	X52815	1	cytoskeletal protein
Atlastin 1 (Atl1)	NM_001009831	1	GTPase, vesicular transport
Bcl-xl	U72350	2	anti-apoptosis
calcyon (Caly)	NM_138915	3	Neuron-specific vesicular protein
Calmodulin (CaM, CALM2)	NM_017326	3	Ca^2+^ binding protein
CD151 antigen (Cd151)	NM_022523	1	Tetraspanin
Choline Kinase β (Chkβ)	NM_017177	3	kinase, phosphocholine biosynthesis
Collapsin Response Mediator Protein-1 (CRMP1)	NM_012932	1	neurite extension, axon guidance
Connective tissue growth factor (CTGF)	NM_022266	1	growth factor
cytochrome c oxidase subunit Vb (Cox5b)	NM_053586	3	energy production
Deoxyhypusine synthase (Dhps)	NM_001004207	1	deoxyhypusine synthase
EH-domain containing 3 (Ehd3)	NM_138890	3	endocytic recycling
Eukaryotic translation elongation factor 1 alpha 1 (Eef1a1)	NM_175838	1	protein synthesis
Lactate dehydrogenase A (LDH-A)	NM_017025	1	glycolytic enzyme
Mortality factor 4 like 2 (Morf4l2)	NM_001007714	2	unknown
Ribosomal protein SA (Rpsa)	NM_017138	2	unknown
tubulin, alpha 1A (Tuba1a)	NM_022298	2	cytoskeletal protein
*Neuroprotective Genes 1-11*			
NPG1, nucleolar protein family 6 (Nol6)	EF688596	1	unknown
NPG2, ADP-ribosylation factor-like 4D, Arl4d	EF688597	1	unknown
NPG3, lysine (K)-specific demethylase 1 (Kdm1)	EF688598	3	histone demethylase
NPG4, MORN repeat containing 4 (Morn4)	EF688599	1	unknown
NPG5, tetraspanin 7 (Tspan7)	EF688600	1	tetraspanin
NPG6, ATPase family, AAA domain containing 1 (Atad1), Thorase	EF688601	1	putative AAA+ ATPase
NPG7, ChaC, cation transport regulator homolog 1 (Chac1)	EF688602	4	unknown
NPG8, ZW10 interactor (Zwint)	EU000468	1	unknown
NPG9, COMM domain containing 9 (Commd9)	EU000469	3	unknown
NPG10, microfibrillar-associated protein 3 (Mfap3)	EU000470	1	unknown
NPG11, 60S ribosomal protein L13a (Rpl13a)	EU000472	1	ribosomal subunit protein
*Genes not tested*			
cytochrome c oxidase subunit III (Co III)	M27315	3	energy production
NPG12, thrombospondin 2, (Thbs2)	EU000471	1	secreted calcium-binding glycoprotein
NPG13, trans-2,3-enoyl-CoA reductase (Tecr)	EU000473	1	unknown

### Functionally cloned genes are neuroprotective

**Figure 3:**
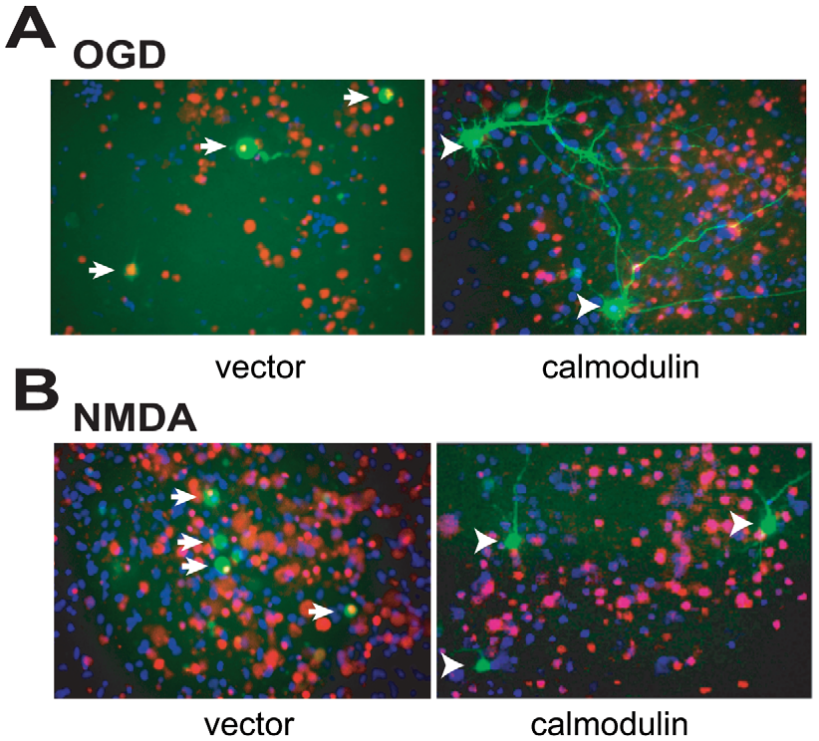
Calmodulin overexpression protects against OGD and NMDA Excitotoxicity. (A and B) Representative photomicrographs of cortical cultures transfected with empty vector or calmodulin and then exposed to (A) 90 min OGD or (B) 500 µM NMDA for 5 min. Cell death was assessed 24 h later. Arrows or arrowheads indicate dead or surviving transfected neurons, respectively.

**Figure 4:**
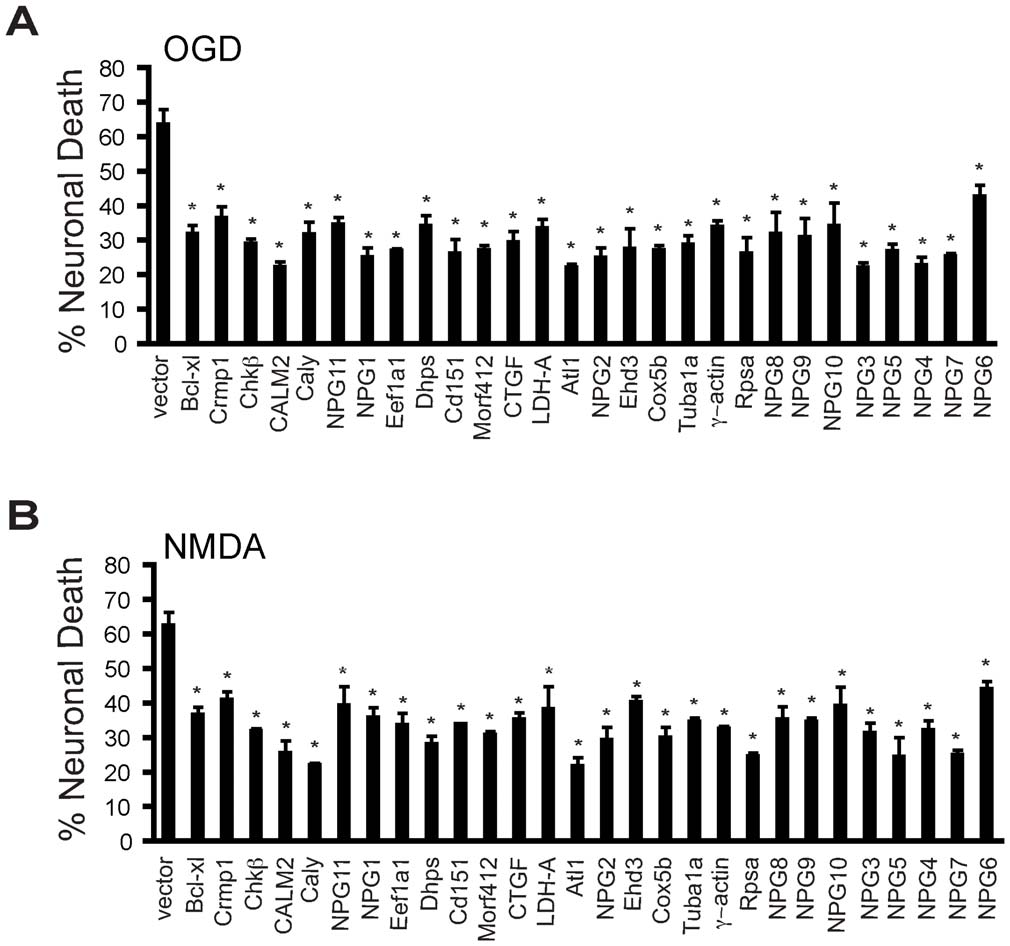
Functionally cloned genes are neuroprotective. (A and B). Primary cortical neurons were transfected with 28 of the identified genes or the empty pCI vector or the ADCGI vector. EGFP serves as the reporter for transfected cells. 48 h later, the neurons were treated with (A) OGD (90 min) or (B) NMDA (500 µM, 5 min). 24 h after treatment, neurons were stained with Hoechst 33342 and propidium iodide. Only EGFP positive cells were scored. Dead neurons were scored as those cells that were propidium iodide positive, with condensed, or fragmented nuclei. Experiments were performed at least three times. Data are the mean ± SEM. *p<0.01 (Student's *t*-test) comparing empty vector to each identified gene.

Of the 31 genes recovered and sequenced, full open reading frames were obtained for 28. To evaluate the neuroprotective actions of the recovered genes, a subset of the full-length genes were subcloned into the multiple cloning site of the mammalian expression vector, ADCGI. The vector is bicistronic vector, in which the cytomegalovirus (CMV) promoter drives the enhanced green fluorescent protein (EGFP) followed by an internal ribosome entry site (IRES) followed by one of identified cloned genes. All the other genes were cloned into the pCI expression vector and primary cortical cultures were transiently transfected with individual genes. When introducing genes into neurons with the pCI vector, pCI-EGFP was co-transfected at ratio of 1∶4 for pCI-EGFP and the pCI-neuroprotective gene, respectively. Forty-eight hours later, the neurons were treated with OGD (90 min) or NMDA (500 µM, 5 min). Twenty-four hours after treatment, the neurons were stained with Hoechst 33342 and propidium iodide. Since only 1–2% of the cells in primary cortical cultures are transfected, only EGFP positive cells were assessed for cell viability. Dead neurons were scored as those cells that displayed propidium iodide positive, condensed or fragmented nuclei (Fig. 3 A, B). All twenty-eight genes tested showed protective effects against both OGD (90 min) (Fig. 4A) and NMDA (500 µM, 5 min) (Fig. 4B) toxicity with relatively similar efficacy. Full open reading frames for three of the clones were not tested, and thus the neuroprotective actions of Co III, NPG12 and NDB13 could not be confirmed as neuroprotective genes.

### Calmodulin and NPG2 participate in preconditioning

**Figure 5:**
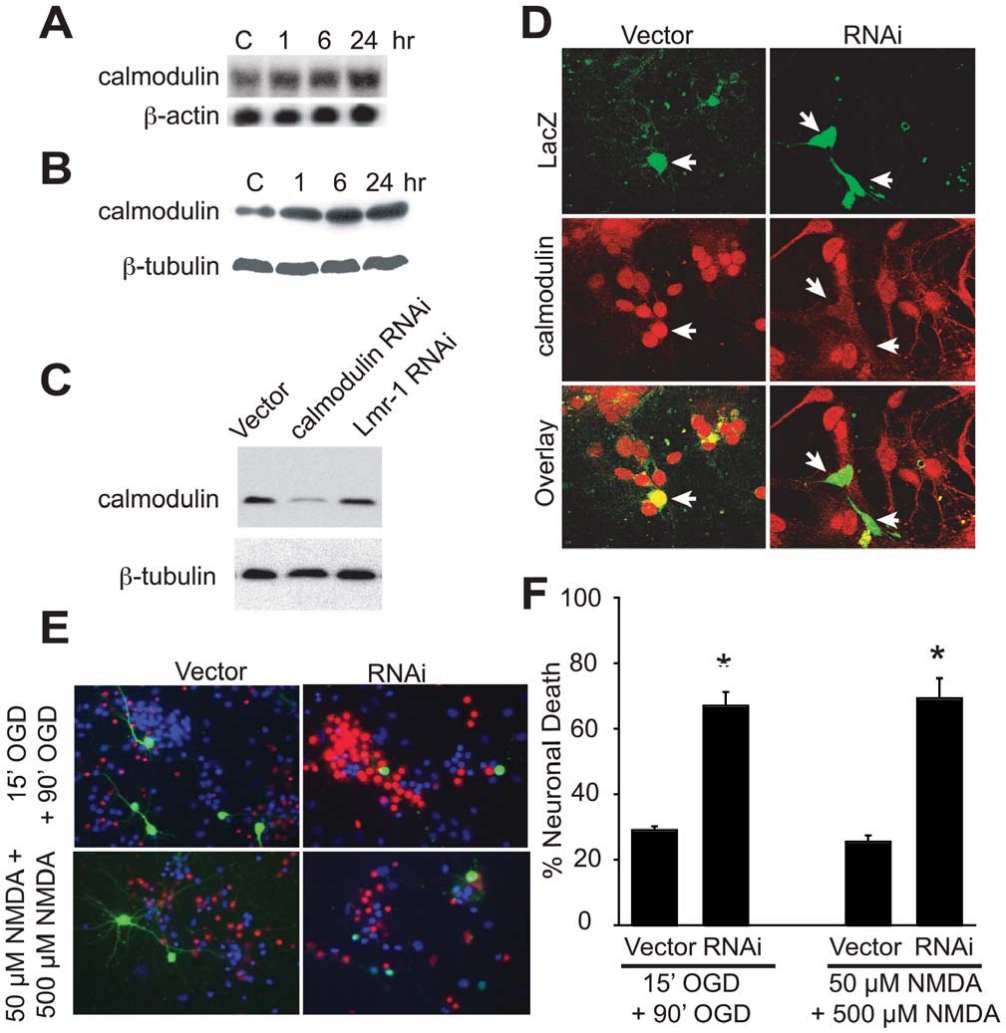
Calmodulin participates in preconditioning induced neuroprotection. Rat primary cortical neurons were treated with 15 min OGD and harvested at the indicated time points for (A) Northern blot or (B) immunoblot analysis. β-actin and β-tubulin are the loading control for Northern blot and immuonblot analysis, respectively. These experiments were conducted 3 times with similar results. (C) HEK 293 cells were transfected with calmodulin RNAi, unrelated RNAi (Lmr-1 RNAi) or empty vector. Two days later Western blot of whole-cell extracts shows reduction of calmodulin expression following calmodulin RNAi treatment but not Lmr-1 RNAi or empty vector. β–tubulin immunoreactivity was used as a loading control. (D) Primary cortical neurons were transfected for 48 h with a control vector or calmodulin RNAi along with CMV-β-Gal. Overlay of the anti-calmodulin and anti-β-Gal antibody staining. White arrows indicate transfected cells. (E and F) Primary cortical neurons were transfected with empty vector or calmodulin RNAi. EGFP serves as the reporter for transfected cells. 48 h later, the cells were preconditioned with OGD (15 min) or NMDA (50 µM, 5 min). 24 h after preconditioning, the cells were treated with OGD (90 min) or NMDA (500 µM, 5 min), respectively. 24 h after treatment, neurons were stained with Hoechst 33342 and propidium iodide. Only EGFP positive cells were scored. Dead neurons were scored as those cells that were propidium iodide positive, with condensed, or fragmented nuclei. (**F**) Quantification of cell viability. Experiments were performed for three times, and the data was presented as mean ± SEM. *p<0.001 (Student's *t*-test) when comparing RNAi to empty vector.

We next evaluated whether the neuroprotective genes identified could be involved in the phenomena of preconditioning. Calmodulin, a gene of known function, and NPG2, a gene of unknown function, were selected for further characterization. In primary cortical neurons exposed to the preconditioning stimulus of 15 min OGD constitutively expressed calmodulin is upregulated at both the mRNA and protein levels (Fig. 5A, B). Because overexpression of calmodulin is neuroprotective against OGD and NMDA, toxicity experiments were conducted to determine if the up-regulation of calmodulin participates in the preconditioning induced neuroprotection. RNAi to calmodulin was designed to knock down the expression of calmodulin. Calmodulin RNAi was introduced into HEK 293 cells and primary cortical neurons via lipofectamine transfection. Knock down of calmodulin in HEK 293 cells was confirmed by immunoblot analysis (Fig. 5C) Transfection with empty vector or an un-related RNAi (Lmr-1 RNAi) had no effect on calmodulin expression showing the specificity of the RNAi to calmodulin (Fig. 5C). Efficacy of knockdown of calmodulin via calmodulin RNAi in cortical neurons was assessed via immunocytochemistry by co-transfecting calmodulin RNAi with β-galactosidase (β-Gal) (Fig. 5D). In cells expressing β-Gal, calmodulin expression is substantially reduced (Fig. 5D). To analyze the effects of calmodulin knock down on the protective effects of preconditioning, primary cortical neurons were co-transfected with calmodulin RNAi and β-galactosidase and compared to empty vector controls prior to the preconditioning exposure. 48 h later, the cells were preconditioned with OGD (15 min) or NMDA (50 µM, 5 min) and 24 hr later the cultures were exposed to a lethal treatment of OGD (90 min) or NMDA (500 µM, 5 min), respectively. 24 h later, the cells were fixed and stained with X-Gal and surviving neurons were scored as those cells that displayed normal morphology. Knocking down calmodulin expression by RNAi treatment during the preconditioning phase is sufficient to block the survival of cortical neurons triggered by OGD or NMDA preconditioning (Fig. 5E, F). These data indicate that calmodulin is a potential important component in the survival response activated by preconditioning.

**Figure 6:**
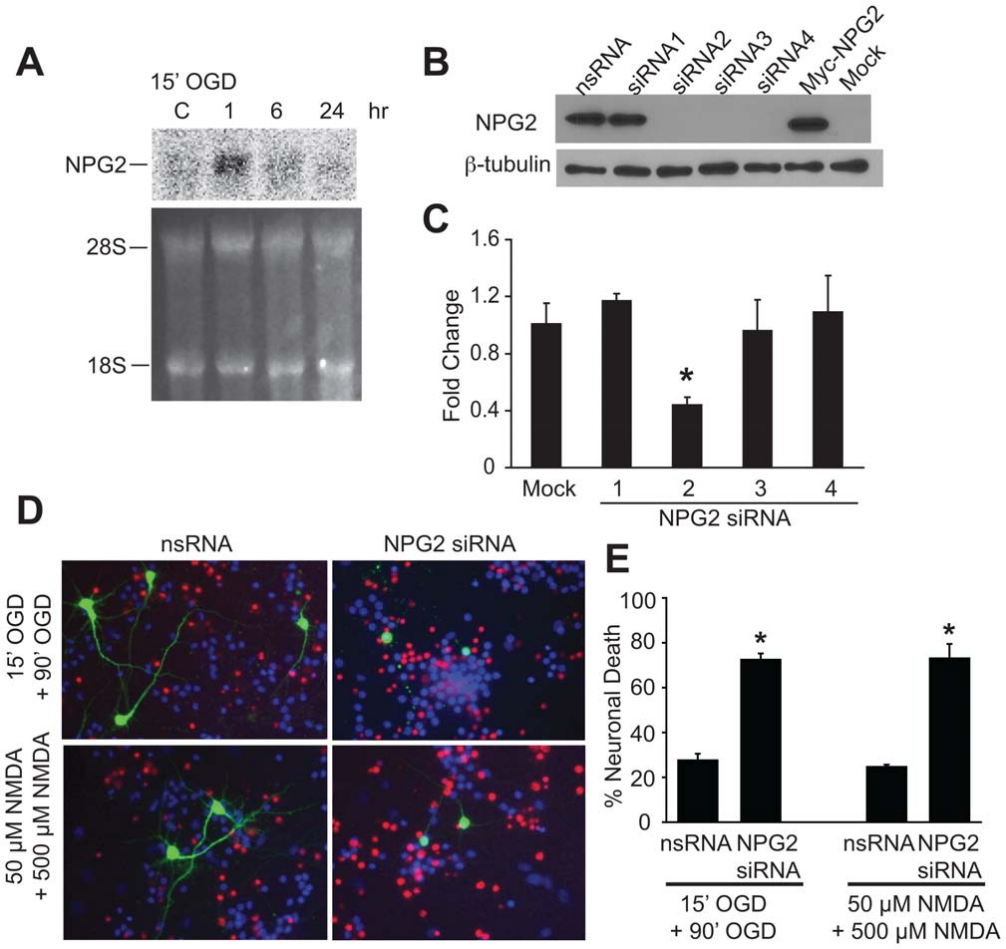
NPG2 participates in preconditioning induced neuroprotection. Primary cortical neurons were treated with 15 min OGD and harvested at the indicated time for (A) Northern blot analysis. Lower panel shows loading of RNA. These experiments were conducted three times with similar results. (B) HEK 293 cells were transfected with myc-tagged NPG2 and non-silencing control siRNA (nsRNA) or four different siRNA to NPG2 (siRNA1-4). Western blots for anti-myc show siRNA 2-4 effectively knock-down NPG2 expression. β–tubulin immunoreactivity was used as a loading control. (C) QT-PCR of Rat-2 cells transfected for 48 h with a control vector or siRNA to NPG2 shows that siRNA2 effectively knocks down endogenous NPG2 transcript. (D and E) Primary cortical neurons were transfected with an empty vector or NPG2 siRNA along with EGFP. 48 h later, the cells were preconditioned with OGD (15 min) or NMDA (50 µM, 5 min). 24 h after preconditioning, the cells were treated with OGD (90 min) or NMDA (500 µM, 5 min), respectively. 24 h later, the cells were stained with Hoescht 33342 and propidium iodide. Only EGFP positive neurons were scored. (D) Viable neurons were scored as those cells that displayed normal morphology. Dead neurons were scored as those cells that were propidium iodide positive, with condensed, or fragmented nuclei. (E) Quantification of cell viability. Experiments were performed three times, and the data was presented as mean ± SEM. *p<0.001 (Student's *t* test) when comparing siRNA to empty vector.

Following exposure to 15 min OGD, NPG2 mRNA expression is induced in primary cortical cultures (Fig. 6A). Four siRNAs to NPG2 were tested for their efficacy in knocking down overexpressed NPG2 epitope tagged with myc at the N–terminus. HEK 293 cell cultures were transfected with the myc-tagged NPG2 in the presences or absence of each of the NPG2 siRNAs. Three out of four NPG2 siRNAs blocked myc-tagged NPG2 expression (Fig. 6B). To test the efficacy of the NPG2 siRNAs to knock down endogenous NPG2, Rat-2 cell cultures were transfected with each of the siRNAs to NPG2 and knock down was assessed by quantitative RT-PCR analysis. Of the four siRNAs, siRNA2 knocked down endogenous NPG2 (Fig. 6C). NPG2 siRNA2 was used to knock down the induction of NPG2 in primary neurons in response to the preconditioning stimulus of 15 min OGD or 50 µM NMDA for 5 min. NPG2 siRNA2 and pCI-EGFP were co-transfected at ratio of 1∶4 for pCI-EGFP and the NPG2 siRNA2, respectively. Scrambled siRNA was used as a control. One day after the preconditioning event the cultures were exposed to lethal OGD or NMDA and 24 hr later, the neurons were stained with Hoechst 33342 and propidium iodide to assess cell viability. Since only 1–2% of the cells in primary cortical cultures are transfected, only EGFP positive cells were assessed for cell viability. Dead neurons were scored as those cells that displayed propidium iodide positive, condensed or fragmented nuclei (Fig. 6D). NPG2 siRNA 2 is sufficient to prevent the neuronal survival induced by OGD and NMDA preconditioning, but scrambled siRNA had no effect (Fig. 6E). These results taken together indicate that NPG2 is a neuroprotective gene that can participate in preconditioning.

## Discussion

Here we describe a functional cloning strategy to identify neuroprotective genes. Thirty-one genes were cloned and identified, and 28 full-length genes were recovered and confirmed to be neuroprotective. This screening method will identify full length genes in the library. It is not a comprehensive screening approach but compared with other screening methods, this functional cloning method has several advantages. Most of current strategies for gene discovery are based on comparison of mRNA changes [4], [5], [6], [7], [8], [9]. Typically the initial screen reveals a very large number, perhaps thousands of mRNA changes, following a single biological manipulation. Most of these changes are not causal to the subsequent phenomena being studied, such as neuroprotection, but are a network response to the exposure of the biologic system to the experimental conditions. It can be tedious to sort through the non-specific response genes to find the critical functional genes. The advantage of the functional screening approach is that the method defines the identification of genes involved in the biologic response of interest, in this case neuroprotection. One of the limitations of the functional screening approach is the size of the full-length inserts in a primary cDNA library. Indeed the majority of the genes identified in this screen were consistent with the average insert size of 2.5 Kb for the library. Now that comprehensive full-length open reading frame libraries are available, additional protective genes could be identified using similar approaches. The power of functional cloning for cytoprotective genes was confirmed by the identification of 31 protective genes. All of the genes protected fibroblasts against oxidative injury induced Parthanatos. Of the 31 genes identified by the functional cloning method, 28 full-length genes were recovered and all provide protection against OGD and NMDA toxicity in primary cortical neurons. Similar studies could be conducted in the future to identify genes that provide protection against other activators of neuronal cell death.

The 31 identified genes fall into various functional categories. Included in this gene set is the well-known protective gene, BCL-xl, which validates the strategy [10]. Calmodulin plays important role in neuronal survival [11]. CTGF promotes mesangial cell survival [12]. Eukaryotic translation elongation factor 1 alpha 1 (Eaf1a1) protects against endoplasmic reticulum stress induced apoptosis [13] and the closely related eEF1A2 may be linked to neurodegeneration [14]. Deoxyhypusine synthase (Dhps) catalyzes the first step in the post-translational modification of a lysine residue in eukaryotic translation initiation factor 5A (eIF5A) to the amino acid hypusine. Dhps may be linked to neuronal survival through eIF5A, since the neuroprotective actions of nerve growth factor are mediated by hypusinated eIF5A [15]. Inhibition of choline kinase is linked to excitotoxic neuronal cell death [13]. Reduced expression of collapsin response mediator protein 1 (CRMP1) promotes the death of spinal cord neurons [16]. Neuroprotective gene 5 (NPG11), also known as thrombospondin 2 (Thbs2) is necessary for synaptic plasticity and functional recovery after stroke [17]. Due to emerging importance of energy metabolism in cell death and survival, genes such as cytochrome c oxidase subunits III and 5b, and lactate dehydrogenase A, are likely to be protective through regulating neuronal energy requirements and maintaining neurons in a bioenergenetically favorable state [18], [19].

Some of the identified genes are linked to neurological disease. Atl1 encodes for the GTPase protein, atlastin. Mutations in Atl1, also known as SPG3A cause autosomal dominant hereditary spastic paraplegia [20]. Mutations in Tuba1 cause abnormal neuronal migration in mice and lissencephaly in humans [21]. NPG5 encodes for a member of the tetraspanin family, tetraspanin 7 (TSPAN7) and mutations in TSPAN7 are linked to forms of X-linked mental retardation in humans [22].

A number of genes without prior connections to survival pathways were identified. Two cytoskeletal proteins, γ-actin and Tuba1a are neuroprotective. CD151 antigen (Cd151) and NPG5, TPAN7 are members of the tetraspanins, which are expressed at high levels in the plasma membrane or in intracellular vesicles. The exact biologic role of tetraspanins is not clear but they may regulate transient cell-to-cell or vesicular communication [23]. Calcyon (Caly) is a neuron-specific vesicular protein that may regulate dopamine receptor signaling [24]. EH-domain containing protein 3 (Ehd3) regulates early endosome to golgi transport [25]. Two ribosomal subunit proteins were identified in the screen including the 40S ribosomal protein SA (Rpsa) and NPG11, also known as 60S ribosomal protein L13a (Rpl13a). NPG3, also known as lysine (K)-specific demethylase 1 (Kdm1) is a histone demethylase [26]. NPG6 is a putative AAA+ ATPase, designated ATPase family, AAA domain containing 1 (Atad1), which we have renamed Thorase. NPG7 is also known as ChaC, cation transport regulator homolog 1 (Chac1). The Chac1 designation is probably a misnomer since NPG7 has weak homology to ChaC, a bacterial protein that is part of the Cha operon. ChaA in bacteria regulates calcium and hydrogen exchange [27], but there is no designated function for ChaB and ChaC. Thus, further study is required to assign a function to NPG7. NPG13, also known as trans-2-3-enoyl-CoA reductase (Tecr) maybe involved in fatty acid elongation [28].

Many of the genes are genes of unknown function including mortality factor 4 like 2 (Morf4l2), NPG1, also known as nucleolar protein family 6 (Nol6), NPG2, also known as ADP-ribosylation factor-like 4D (ARl4d), NPG4, also known as MORN repeat containing 4 (Morn4), NPG8, also known as ZW10 interactor (Zwint), NPG9, also known as COMM domain containing 9 (Commd9) and NPG10, also known as NPG10, microfibrillar associated protein 3 (Mfap3). How each of the proteins of known function and unknown function regulate cell survival will require further study, but the diversity of proteins identified in this functional screen for protective proteins indicates that there are many intrinsic cell survival pathways.

Since all the protective genes identified in this study derived from a preconditioned library, we explored the role of two of the genes in preconditioning induced neuronal survival. Knockdown of either calmodulin or NPG2 eliminates the protection induced by preconditioning. Whether any of the other protective genes identified here play a role in preconditioning is not known and requires further study. The fact that two seemingly unrelated genes are sufficient for neuronal protection after preconditioning suggests that there are either unrelated but essential pathways for preconditioning induced neuronal survival or preconditioning induced neuronal survival is due a complex set of co-induced proteins, which are essential for the survival response. These data suggest that there is a complex epistasis where multiple parallel pathways are capable of producing a neuroprotective phenotype with perhaps some convergent targets. As technology for genome-wide detection of transcriptional targets becomes more robust, we will be in a better position to address these points directly.

In summary, we have identified a broad range of genes that are neuroprotective when overexpressed. For some genes identified there are known biologic actions that would support a neuroprotective role. Most of the genes indentified here have, as of yet, unexplored roles in regulating neuronal survival and death. Further study of these genes holds tremendous opportunity to identify heretofore unexplored modes of neuronal survival and death and offers opportunities for new neuroprotective strategies in the brain.

## Materials and Methods

### Ethics Statement

This study was performed in accordance with the recommendations in the Guide for the Care and Use of Laboratory Animals of the National Institutes of Health. The protocol for generation of primary neuronal cultures was approved by the Johns Hopkins Animal Care and Use Committee under protocol number MO09M136.

### Primary Neuronal Cultures

Primary neuronal cortical cultures were generated as described [29]. Briefly, embryonic day 16 rat cortex was dissected under a microscope, exposed for 20 min to a 0.027% trypsin/saline solution [5% PBS (in mM) 40 sucrose, 30 glucose, 10 HEPES, pH 7.4], and then dissociated in modified Eagle's medium (MEM), 10% horse serum, 10% fetal bovine serum, and 2 mM glutamine, by trituration. The cells were plated on 15 mm multiwell plates (NUNC, Rochester, NY) coated with poly-ornithine (Invitrogen, Carlsbad, CA) at a density of 3–4×10^5^ cells per well. Four days after plating the cells were treated with 5-fluoro-2-deoxyuridine (Sigma, St. Louis, MO) for three days to inhibit proliferation of non-neuronal cells. Cultures were maintained in MEM, 5% horse serum, and 2 mM glutamine in 7% CO_2_, in a humidified 37°C atmosphere. Mature neurons (12–14 d in culture) were used for all experiments. In mature cultures, neurons represent 70–90% of the total number of cells.

### OGD Preconditioning and OGD Toxicity

Combined oxygen glucose deprivation (OGD) was performed in mature cortical neurons as described [30] by complete exchange with deoxygenated, glucose-free controlled salt solution (CSS) containing: 120 mM NaCl, 5.4 mM KCl, 1.8 mM CaCl_2_, 25 mM Tris-HCl, bubbled with 10% H_2_/85% N_2_/5% CO_2_. The cultures were kept in an anaerobic chamber containing the gas mixture, 10% H_2_/85% N_2_/5% CO_2,_ for 15 and/or 90 min at 37°C. Combined OGD was terminated by replacement with oxygenated growth media. Cell death assessments were performed 24 h after the lethal 90 min OGD challenge.

### NMDA Excitotoxicity

Neuronal cultures were exposed to 5 min of 500 µM NMDA (Sigma, St. Louis, MO) with 10 µM glycine (Fisher, Pittsburgh, PA) to induce excitotoxicity as described [29]. Exposure was terminated by replacement with growth media. Cell death assessments were performed 24 h later.

### NMDA Preconditioning and Excitotoxicity

To induce preconditioning with NMDA the cells were exposed to 5 min of 50 µM NMDA/10 µM glycine in CSS. The treatment was terminated by wash and full exchange into growth media. 24 hr later the culture were exposed to 5 min of 500 µM NMDA/10 µM glycine to induce excitotoxicity. Exposure was terminated by replacement with growth media.

### cDNA library

Rat primary cortical neurons were preconditioned with 15 min OGD. 16 hours later, mRNA was isolated from the preconditioned neurons with TRIzol reagent (Invitrogen, Carlsbad, CA) per the manufacturers instructions. The isolated mRNA was used to make double-strand cDNA using the SuperScript Kit (Invitrogen, Carlsbad, CA) per the manufacturers instruction. The cDNAs were cloned into pFB retroviral vector per the manufacturers instructions (Stratagene,La Jolla, CA).

### Retrovirus production and fibroblast infection

Plasmids (3×10^6^) containing the OGD preconditioned cDNA library were transfected into Phoenix amphopack 293 cells (Dr. Garry Nolan, Stanford University) [31] with Lipofectamine 2000. The cells were allowed to grow another 48 hours. The culture media containing retrovirus was collected, filtered (0.45 µM filter [Corning Incorporated, Lowell, MA]) and diluted 1∶10 or 1∶100 in fibroblast growth media (Dulbecco's modified Eagle's medium [DMEM], 10% fetal bovine serum) supplemented with polybrene (8 µg/ml) (Sigma, St. Louis, MO). Fibroblasts were infected with 100 µl of retrovirus media that was added to mouse fibroblasts plated at 800 cells/well of a 96 well plate (NUNC, Rochester, NY). 24 h later the culture media was fully exchanged with fibroblast growth media. Fibroblasts were maintained in fibroblast growth media, 7% CO_2_, humidified 37°C atmosphere.

### Functional screening and recovery of cytoprotective genes

48 h after retroviral infection of mouse fibroblasts, they were treated with menadione (175 µM, 10 min). Surviving cells were grown and amplified another 9–13 days. A fraction of these cells were treated again with menadione to eliminate false positives. To recover the cytoprotective genes, PCR with oligonucleotide primers (Forward 5′-GGC TGC CGA CCC CGG GGG TGG-3′, Reverse 5′-CGA ACC CCA GAG TCC CGC TCA-3′) that recognize regions flanking the multiple cloning site of the pFB vector was used to recover the cDNA from genomic DNA. The PCR products were cloned into PCR-xl-TOPO cloning vector (Invitrogen, Carlsbad, CA) by the method provided with the kit and sequenced by the Genetic Core sequencing facility at the Johns Hopkins University School of Medicine.

### Cytotoxicity Assessment

Assessment of the protective effects of 15 min OGD preconditioning against lethal 90 min OGD was performed by staining with 1 µg/ml Hoechst (Sigma, St. Louis, MO) and 7 µM propidium iodide (Sigma, St. Louis, MO) and unbiased computer assisted cell counting [29]. Assessment of the potential protective effects of the neuroprotective genes was performed by transfecting primary cortical neurons with the identified gene or the empty vector (pCI with EGFP). EGFP served as the reporter for transfected cells. 48 h later, the neurons were treated with OGD (90 min) or NMDA (500 µM, 5 min). 24 h after treatment, neurons were stained with 1 µg/ml Hoechst 33342 and 7 µM propidium iodide. Only EGFP positive cells were scored. Dead neurons were scored as those cells that displayed PI positive, condensed, or fragmented nuclei. At least three separate experiments using 2 separate wells were performed for a minimum of 100 neurons counted for each well. Fibroblast survival was assessed 24 h after treatment and the viability was determined by cell morphology. Live cells were scored as those displayed normal morphology and dead fibroblasts were scored as those cells that were shrunken and round in shape.

### Expression Vectors and Transfection of Cultured Neurons

Expression vector pCI vector was used for the expression of identified genes. The pCI vector (Promega, San Luis Obispo, CA) has the gene of interest under the control of the CMV promoter. A subset of the full-length genes were subcloned into the multiple clone site of a mammalian expression vector (ADCGI vector). The vector is bicistronic vector, which the cytomegalovirus (CMV) promoter drives the enhanced green fluorescent protein (EGFP) followed by internal ribosome entry site (IRES) promoter and then followed by cloned gene. Primary cortical neurons were maintained in culture for 12 days before transfection via Lipofectamine 2000 (Invitrogen, Carlsbad, CA) according to manufacturer instructions. The PCI vector containing the gene of interest was transfected at 0.8 µg/well together with pCI-EGFP at 0.2 µg/well per 24 well plate. 48 h after transfection, neurons were challenged with either 90 min OGD or 500 µM NMDA.

### Full Length Clones

Bcl-xl, Eef1a1, CD151 antigen, LDH-A, Cox5b, Tuba1 Chkβ, Calmodulin, Calcyon, Dhps, Morf4l2, Spg3a, Arf4l_predicted, Rpsa, Tm4sf2, 531-7, 547-6 and Mfap3 full length clones were obtained during the PCR recovery of neuroprotective genes and were subsequently cloned into the pCI expression vector using the multiple cloning site. Truncated forms of all the other genes were obtained during the PCR recovery and such full-length mouse, rat or human clones were purchased for further study. Mouse CRMP1, rat Rpl13a, mouse Nol6, mouse CTGF, rat Zwint-1, mouse AOF2 full-length clones were obtained from Open Biosystems, Alabama, and were cloned in pCI vector for expression. Homo sapiens Ehd3 was obtained from Dr. David N. Louis, Harvard Medical School [32], and was cloned in pCI vector for expression. The rat 697-27 full-length clone was obtained by screening of full-length library [32] with the truncated 697-27 PCR probe, and it was cloned into the pCI vector for expression.

### Northern Blot Analysis

Total RNA (10 µg) was separated on 1% agarose gels containing 2.2 M formaldehyde, transferred to a nylon membrane (Hybond-XL, Amersham, Piscataway, NJ) and fixed. Probes were made from cDNA sequences and labeled with α-^32^P dATP using a random primer labeling kit (Amersham, Piscataway, NJ). Membranes hybridized washed and were exposed to a PhosphorImager plate for 1 day. The bands were visualized and quantified using a PhosphorImager (Molecular Dynamics, Sunnyvale, CA). The blots were stripped and rehybridized with a ^32^P-labeled mouse β-actin probe to assess RNA loading efficiency.

### RNAi and siRNA

Two complementary short oligo DNA (forward 5′-CCC TGA ATT TCT GAC AAT GTT CAA GAG ACA TTG TCA GAA ATT CAG GGT TTT TT-3′ and reverse 5′-AAT TAA AAA ACC CTG AAT TTC TGA CAA TGT CTC TTG AAC ATT GTC AGA AAT TCA GGG GGCC-3′) were synthesized according to the calmodulin sequence. Double stranded DNA was made by denaturing at 95°C for 5 min then renaturing at 37°C for 1 hr. The double stranded DNA was cloned into the gene silencer vector (Ambion, Austin, TX).

#### siRNA

Four Arf4l siRNAs (siRNA1: sense 5′-GACUGGAGACAUAUGUAAAdTdT-3′ and antisense 5′-UUUACAUAUGUCUCCAGUCdTdT-3′; siRNA2: sense 5′-GGAAGACUUCCCUUCUUUAdTdT-3′ and antisense 5′-UAAAGAAGGGAAGUCUUCCdCdG-3′; siRNA3: sense 5′-AGACUGGAGACAUAUGUAAdTdT-3′ and antisense 5′-UUACAUAUGUCUCCAGUCUdTdG-3′; siRNA4: sense 5′-GCCUCAAGUUCAAGGAAUUdTdT-3′ and antisense 5′-AAUUCCUUGAACUUGAGGCdGdG-3′) and scramble control siRNA (sense 5′-UUCUCCGAACGUGUCACGUdTdT-3′ and antisense 5′-ACGUGACACGUUCGGAGAAdTdT-3′) were ordered from Qiagen (Valencia, CA). siRNAs were introduced into cells with GeneSilencer transfection reagent (Gene Therapy Systems, San Diego, CA) or lipofectamine transfection reagent (Invitrogen, Carlsbad, CA).

### Immunoblotting

Cell cultures following the different treatment paradigms were collected in 100 µl of Tris HCl buffer (pH 7.4) containing 2 mM EDTA, 1 mM β-mercaptoethanol, 1 mM PMSF, 1 mM benzamide and leupeptin (10 µl/ml). Equal protein was loaded per lane into a 12% polyacrylyamide gel. After electrophoresis and nitrocellulose membrane (Bio-Rad, Hercules, CA) transfer, the membranes were incubated overnight with antibodies. The primary antibody (anti-calmodulin, Abcam, Cambridge, MA; anti-myc) were diluted 1∶1000 in 5% dry nonfat milk, 0.1% Tween-20 in phosphate buffer (pH 7.4). Subsequently, nitrocellulose membranes were incubated for 3 hr with secondary antibody (1∶5000) (Pierce, Rockford, IL) at room temperature in 5% non-fat dry milk in phosphate buffer (pH 7.4). The labeled bands were visualized using chemiluminescence (Pierce, Rockford, IL).

### Immunohistochemistry

Cells were washed with PBS and fixed in fresh 4% paraformaldehyde at 4°C for 1 hr, then blocked with 4% normal goat serum (Abcam, Cambridge, MA) in PBS for 30 min at room temperature. Antibody (LacZ antibody was from Sigma, St. Louis, MO; Calmodulin antibody was from Abcam, Cambridge, MA) was applied for 12–16 hr at 4°C. After two 10 min wishes with PBS, cultures were incubated in fluorescent FITC or rhodamine conjugated secondary antibody (Abcam, Cambridge, MA) for 1 hr at room temperature, and then washed again with PBS as before. The cultures were incubated with nucleus staining dye TOTO-3 (1∶1000) (Applied Biosystems, Foster City, CA) for 10 min at room temperature, and then washed twice (5 min) with PBS.

### siRNA

Four Arf4l siRNAs (siRNA1: sense 5′-GAC UGG AGA CAU AUG UAAAdTdT-3′ and antisense 5′-UUU ACA UAU GUC UCC AGUCdTdT-3′; siRNA2: sense 5′-GGA AGA CUU CCC UUC UUUAdTdT-3′ and antisense 5′-UAA AGA AGG GAA GUC UUC CdCdG-3′; siRNA3: sense 5′-AGA CUG GAG ACA UAU GUAAdTdT-3′ and antisense 5′-UUA CAU AUG UCU CCA GUC UdTdG-3′; siRNA4: sense 5′-GCC UCA AGU UCA AGG AAU UdTdT-3′ and antisense 5′-AAU UCC UUG AAC UUG AGG CdGdG-3′) and non-silencing control siRNA (sense 5′-UUC UCC GAA CGU GUC ACGUdTdT-3′ and antisense 5′-ACG UGA CAC GUU CGG AGAAdTdT-3′) were ordered from Qiagen (Valencia, CA).

### Quantitative RT-PCR

10 ng total RNA was assayed by quantitative RT-PCR with one step QuantiTect SYBR Green RT-PCR Kit (Qiagen, Valencia, CA) on an ABI 7900HT real-time PCR machine. Reaction was performed in a 20 µL reaction mixture consisting of 10 µL 2× QuantiTect SYBR Green, 10 ng RNA, 0.2 µL QuantiTect RT mix and 0.6 µmol/L of each primer. NPG2 (forward 5′-AAG GAC TGA GGG AGC ATA-3′ and reverse 5′-.TAG GTG AAG GGC AGT GAA GA-3′). Reverse transcription was performed at 50°C for 30 min. Quantitative RT-PCR was run for 40 cycles (94°C for 15 sec, 55°C for 30 sec, and 72°C for an additional 30 sec) following a hot start (95°C for 10 min). Melting curves were obtained by increasing the temperature from 55°C to 96°C in increments of 0.5°C and samples were examined for the specificity of the PCR products. Each sample was quantified by determining the cycle threshold (Ct) and triplicate qRT-PCR reactions were done for each mRNA samples. Glyceraldehyde-3-phosphate dehydrogenase (GAPDH) was used as the reference gene.

### Statistical Analysis

For all quantitative data, significant overall F values were obtained by using a one-way, between-groups analysis of variance (ANOVA). Specific comparison on all possible pair wise combinations were made with the Student's *t*-test for independent means. Significance was determined at p<0.05.
